# Effectiveness of High-Fiber, Plant-Based Diets in Reducing Cardiovascular Risk Factors Among Middle-Aged and Older Adults: A Systematic Review

**DOI:** 10.7759/cureus.67660

**Published:** 2024-08-24

**Authors:** Manorama Pandey, Osamah AlQassab, Tatchaya Kanthajan, Aneri Parikh, Aida J Francis, Chithra Sreenivasan, Marcellina Nwosu

**Affiliations:** 1 Internal Medicine, California Institute of Behavioral Neurosciences and Psychology, Fairfield, USA; 2 Clinical Research, California Institute of Behavioral Neurosciences and Psychology, Fairfield, USA

**Keywords:** plant-based diets, dietary fibers, soluble fiber, insoluble fiber, cardiovascular risk factors, older adults, middle-aged adults

## Abstract

Cardiovascular disease (CVD) is a prominent contributor to morbidity and mortality, particularly in the middle-aged and elderly population. Plant-based, high-fiber diets high in fruits, vegetables, whole grains, legumes, and nuts can significantly lower CVD risk factors. This systematic review aims to assess how effectively diet improves cardiovascular health in this demographic. Using the Preferred Reporting Items for Systematic Reviews and Meta-Analyses (PRISMA) criteria, we thoroughly searched PubMed, Google Scholar, ScienceDirect, Cochrane Library, and ClinicalTrials.gov, explicitly focusing on papers published in English. The review identified 10 pertinent papers, including three systematic reviews, one randomized-controlled trial (RCT), two observational studies, and four review articles demonstrating significant improvements in blood pressure, cholesterol levels, and glycemic management associated with high-fiber plant-based diets (PBDs). The research specifically emphasized the significance of dietary fiber in decreasing low-density lipoprotein (LDL) cholesterol, enhancing insulin sensitivity, and reducing systemic inflammation. These data support the concept that PBDs high in fiber can effectively lower CVD risk factors. However, limitations such as self-reported dietary intake and variability in adherence were noted. In conclusion, high-fiber PBDs are a viable strategy for managing and preventing CVD in middle-aged and older adults. Future research should focus on long-term adherence, the comparative benefits of different plant-based foods, and developing personalized dietary recommendations to optimize cardiovascular health outcomes in this population.

## Introduction and background

Cardiovascular disease (CVD) remains the leading cause of death worldwide, accounting for one in every three deaths globally [1]. Modifiable lifestyle factors, including smoking, excessive consumption of animal fats and added sugars, and physical inactivity, significantly contribute to the prevalence of heart disease. Plant-based diets (PBDs), which emphasize cereals, fruits, vegetables, legumes, and nuts while restricting animal-based products, have gained increasing interest among the general population and the scientific community for their potential health benefits [2]. These diets differ in the extent to which they limit the use of animal products: ovo-vegetarians consume eggs while abstaining from other animal products; lacto-ovo vegetarians include dairy and eggs in their diet but avoid meat; pescatarians typically consume plant-based foods but occasionally include fish and dairy; vegans, on the other hand, omit all animal products from their diet [3,4].

Dietary fibers, both soluble and insoluble, are crucial components of PBDs. Insoluble fibers, like cellulose, some hemicelluloses, and lignin, and soluble fibers, such as pectin, gums, and mucilage derived from psyllium husk and β-glucan, play essential roles in gastrointestinal health [5]. The American Heart Association recommends consuming 25-30 grams of fiber daily to maintain optimal health. Yet, the reality in many Western countries falls short, with the average daily intake hovering around 15 grams. This fiber deficit is not just a nutritional gap - it's a potential health hazard [6,7]. Insufficient fiber intake is closely linked to various health problems, from cardiovascular events to metabolic disorders. Insoluble fibers increase gut transit time, while gut microbiota ferments prebiotic fibers to produce short-chain fatty acids (SCFAs), like butyrate, which promote gastrointestinal health and regulate the immune system [8]. A reduction in butyrate and SCFA-producing bacteria is associated with gut dysbiosis and hypertension [9].

Clinical trials have demonstrated that adopting a low-fat, animal product-free diet, regular physical activity, and effective stress management can not only halt but potentially reverse the buildup of atherosclerotic plaque. Embracing vegetarian diets has been associated with a significantly lower risk of developing CVD, ischemic coronary artery disease, and cerebrovascular disorders, highlighting their powerful protective benefits [10]. Individuals following vegetarian diets often exhibit fewer heart disease risk factors, such as high cholesterol and hypertension [11,12]. PBDs are cardioprotective due to their low saturated fat content and lack of harmful substances, including nitrosamines, heme iron, and advanced glycation end products found in meat. Fiber from whole grains is particularly beneficial for cardiovascular health [13]. Gut microbes metabolize heme iron and compounds like choline and L-carnitine in animal products to produce trimethylamine-N-oxide (TMAO), which promotes atherosclerosis and increases CVD risk [14,15]. Despite these promising findings, the specific impact of high-fiber PBDs on CVD risk factors among middle-aged and older adults requires further investigation.

This systematic review thoroughly assesses the effectiveness of high-fiber PBDs in reducing CVD risk factors among middle-aged and older adults. It analyzes data from multiple studies to offer practical insights into the effects of dietary interventions in this specific population. The goal is to empower doctors, researchers, and healthcare practitioners to make informed recommendations based on solid evidence, thereby contributing to the improvement of cardiovascular health.

## Review

Methods

This review substantially adhered to the Preferred Reporting Items for Systematic Reviews and Meta-Analysis (PRISMA) standards [16].

Research objective

This systematic review examined various studies to evaluate the effectiveness of high-fiber PBDs in decreasing CVD risk factors in middle-aged and older individuals. The primary objective was to assess the effects of these dietary changes on blood pressure, cholesterol levels, glycemic management, and patient-reported outcomes. The aim is to provide helpful insights to support evidence-based decision-making and to recognize potential research gaps when managing CVD risk through dietary interventions.

Inclusion and exclusion criteria

The study focuses on research involving middle-aged (45-65 years) and older adults (65 years and above) diagnosed with obesity or overweight and at least one of the following conditions: type 2 diabetes mellitus (T2DM), ischemic heart disease, hypertension, or hypercholesterolemia. It includes systematic reviews, randomized-controlled trials (RCTs), observational studies, and review articles that examine the effects of whole food plant-based (WFPB) diets, documenting changes in body mass index (BMI), cholesterol levels, blood pressure, glycemic management, patient-reported outcomes, and any negative consequences associated with dietary treatments. Only studies published in English within the past decade were considered to ensure relevance and accuracy. Studies such as case reports, case series, and those focusing on non-cardiovascular health outcomes were excluded due to their limited capacity to offer strong evidence.

Search strategy

We performed an extensive literature search by utilizing five electronic databases: PubMed, Google Scholar, ScienceDirect, Cochrane Library, and ClinicalTrials.gov. Medical Subject Headings (MeSH) were created and combined with specific keywords using a MeSH strategy. We then integrated the MeSH terms and keywords for the five concepts using AND for an advanced search strategy. The detailed search strategy, including the combined MeSH terms and keywords, is provided in Table 1.

**Table 1:** Search strategy using MeSH terms and keywords MeSH: Medical Subject Headings

Concepts	Keywords	Combined MeSH with keywords using OR
High-fiber diet	High-fiber diet	High-fiber diet OR ("Dietary Fiber/administration and dosage"[Majr] OR "Dietary Fiber/adverse effects"[Majr] OR "Dietary Fiber/classification"[Majr] OR "Dietary Fiber/deficiency"[Majr] OR "Dietary Fiber/history"[Majr] OR "Dietary Fiber/metabolism"[Majr] OR "Dietary Fiber/pharmacology"[Majr] OR "Dietary Fiber/poisoning"[Majr] OR "Dietary Fiber/radiation effects"[Majr] OR "Dietary Fiber/supply and distribution"[Majr] OR "Dietary Fiber/therapeutic use"[Majr] OR "Dietary Fiber/toxicity"[Majr])
Plant-based diet	A plant-based diet, vegetarian diet, vegan diet	Plant-based diet OR vegetarian diet OR vegan diet OR ("Diet, Plant-Based/adverse effects"[Majr] OR "Diet, Plant-Based/classification"[Majr] OR "Diet, Plant-Based/ethics"[Majr] OR "Diet, Plant-Based/history"[Majr] OR "Diet, Plant-Based/mortality"[Majr] OR "Diet, Plant-Based/psychology"[Majr] OR "Diet, Plant-Based/trends"[Majr])
Cardiovascular risk factors	Cardiovascular risk factors, heart disease risk factors, cardiometabolic risk factors, atherosclerotic risk factors, coronary artery disease risk factors, hypertension risk factors, dyslipidemia risk factors	Cardiovascular risk factors OR heart disease risk factors OR cardiometabolic risk factors OR atherosclerotic risk factors OR coronary artery disease risk factors OR hypertension risk factors OR dyslipidemia risk factors OR ("Hypertension/complications"[Majr] OR "Hypertension/diet therapy"[Majr] OR "Hypertension/drug therapy"[Majr] OR "Hypertension/epidemiology"[Majr] OR "Hypertension/genetics"[Majr] OR "Hypertension/mortality"[Majr] OR "Hypertension/physiopathology"[Majr] OR "Hypertension/prevention and control"[Majr]) OR ("Hyperlipidemias/diet therapy"[Majr] OR "Hyperlipidemias/etiology"[Majr] OR "Hyperlipidemias/mortality"[Majr] OR "Hyperlipidemias/physiopathology"[Majr] OR "Hyperlipidemias/prevention and control"[Majr] OR "Hyperlipidemias/psychology"[Majr]) OR ("Diabetes Mellitus/diet therapy"[Majr] OR "Diabetes Mellitus/etiology"[Majr] OR "Diabetes Mellitus/mortality"[Majr] OR "Diabetes Mellitus/physiopathology"[Majr] OR "Diabetes Mellitus/prevention and control"[Majr]) OR ("Smoking/adverse effects"[Majr] OR "Smoking/history"[Majr] OR "Smoking/metabolism"[Majr] OR "Smoking/mortality"[Majr] OR "Smoking/physiopathology"[Majr] OR "Smoking/psychology"[Majr] OR "Smoking/therapy"[Majr] OR "Smoking/trends"[Majr]) OR ("Obesity/classification"[Majr] OR "Obesity/diet therapy"[Majr] OR "Obesity/etiology"[Majr] OR "Obesity/mortality"[Majr] OR "Obesity/physiopathology"[Majr] OR "Obesity/prevention and control"[Majr] OR "Obesity/therapy"[Majr])
Middle-aged adults	Middle-aged adults	Middle-aged adults OR ("Middle Aged/physiology"[Majr] OR "Middle Aged/psychology"[Majr])
Older adults	Older adults	Older adults OR ("Aged/physiology"[Majr] OR "Aged/psychology"[Majr] OR "Aged/statistics and numerical data"[Majr])

Data extraction

Data extraction encompassed the gathering of information about various aspects of the study, including study particulars (such as authors, publication year, and study design), participant characteristics (such as age, gender, and health status), intervention specifics (such as the type of high-fiber PBD, duration, and frequency), details regarding the comparison group, and outcome data (including the average change in CVD risk factors, such as blood pressure, cholesterol levels, and glycemic control, as well as enhancements in symptoms and quality of life).

Quality assessment

The set of 10 incorporated research studies included three systematic reviews, one RCT, four literature review publications, and two observational studies. We evaluated the quality of these studies using specific assessment tools: the Assessment of Multiple Systematic Reviews (AMSTAR) tool for systematic reviews, the Cochrane Risk of Bias Assessment tool for RCTs, the Scale of the Assessment of Narrative Review Articles (SANRA) checklist for evaluating narrative review articles, and the Newcastle-Ottawa Scale (NOS) for observational research. The detailed quality assessment results are provided in Tables 2-5.

**Table 2:** Quality appraisal using Assessment of Multiple Systematic Reviews (AMSTARs) PICO: Population, Intervention, Comparison, and Outcomes

	Gan et al. (2021) [17]	Austin et al. (2021) [18]	Yao et al. (2023) [19]
Were the research questions and inclusion criteria for the review designed to encompass all elements of PICO?	Yes	Yes	Yes
Does the report clearly explain that the review methodologies were defined prior to the review and provide a justification for any significant deviations from the protocol?	Yes	Partial yes	Yes
Were the study designs chosen by the review authors justified?	Yes	Yes	Yes
Did the writers of the review employ a comprehensive and meticulous methodology for conducting a literature search?	Yes	Yes	Yes
Were the review authors able to replicate the study selection process?	Yes	Yes	Yes
Did the review writers conduct data extraction in duplicate?	Yes	Yes	Yes
Did the authors of the review provide an all-encompassing list of papers that were omitted and offer a justification for their exclusion?	No	No	No
Did the reviewers adequately describe the studies that were included?	Yes	Yes	Yes
Did the authors of the review apply an appropriate strategy to assess the risk of bias in the individual studies included in the review?	No	No	No
Did the authors of the review include details regarding the funding sources for the research incorporated in the review?	Yes	Yes	Yes
When combining the statistical data for the meta-analysis, did the review writers adhere to the appropriate methods?	Not applicable	Not applicable	Not applicable
Did the reviewers consider the potential impact of bias risk in individual studies on the results of the meta-analysis or any other evidence synthesis if one was performed?	Not applicable	Not applicable	Not applicable
Did the authors of the review consider the risk of bias in individual studies when analyzing and explaining the conclusions of the review?	Yes	Yes	Yes
Did the review authors offer an adequate explanation and justification for any discernible disparities in the results?	Yes	Yes	Yes
Did the authors of the review do a comprehensive evaluation of publication bias (also known as small study bias) and acknowledge its potential impact on the review's results if they conducted a quantitative synthesis?	Not applicable	Not applicable	Not applicable
Did the review authors provide any information about potential conflicts of interest, such as any financial support they received for the review?	Yes	Yes	Yes

**Table 3:** Quality Appraisal using the Cochrane Risk-of-Bias assessment tool + (positive): The study incorporated the criterion; - (negative): The study omitted the criterion.

	Wright et al. (2017) [20]
An allocation sequence was constructed with sufficient adequacy.	+
The allocation sequence was effectively concealed.	+
The distribution was effectively hidden.	+
The intervention was not communicated to the participants and staff.	-
Reviewers of the outcomes are uninformed about the intervention.	+
The issue of incomplete outcome data was effectively resolved.	+
The study reports did not show any signs of selective outcome reporting.	+
The study lacked any other factors that could compromise its objectivity.	+

**Table 4:** Quality appraisal using the Scale for the Assessment of Narrative Review Articles (SANRAs) 0: Low standard (the study did not meet the required criterion); 1: Intermediate standard (the study only fulfilled some of the criteria); 2: High standard (the study met the criteria)

	Soliman (2019) [21]	Nepali et al. (2022) [22]	Thomas et al. (2023) [23]	Salehin et al. (2023) [24]
The article's significance was justified.	2	2	2	2
Specific objectives or the formulation of inquiries were established.	2	1	1	2
A description of the literature search was provided.	1	0	1	2
The citation was completed.	2	2	2	2
The process of reasoning was based on evidence.	2	2	2	2
The information was presented appropriately.	2	2	2	2

**Table 5:** Quality appraisal using the Newcastle-Ottawa Quality Assessment Scale (NCOS) 0: The study did not incorporate the criterion; 1: The study incorporated the criterion

	Zhang et al. (2022) [25]	Shivakoti et al. (2022) [26]
Representativeness of the exposed cohort	1	1
Selection of the nonexposed cohort	1	1
Ascertainment of exposure	1	1
Evidence indicating that the desired result was absent at the beginning of the investigation.	1	1
Ensuring the comparability of cohorts based on their design or analysis	1	1
Assessment of outcome	1	1
Was the duration of the follow-up sufficient for the results to appear?	1	1
Sufficient cohort follow-up	1	1
Total score	8	8

Results

Study Selection

**Figure 1:**
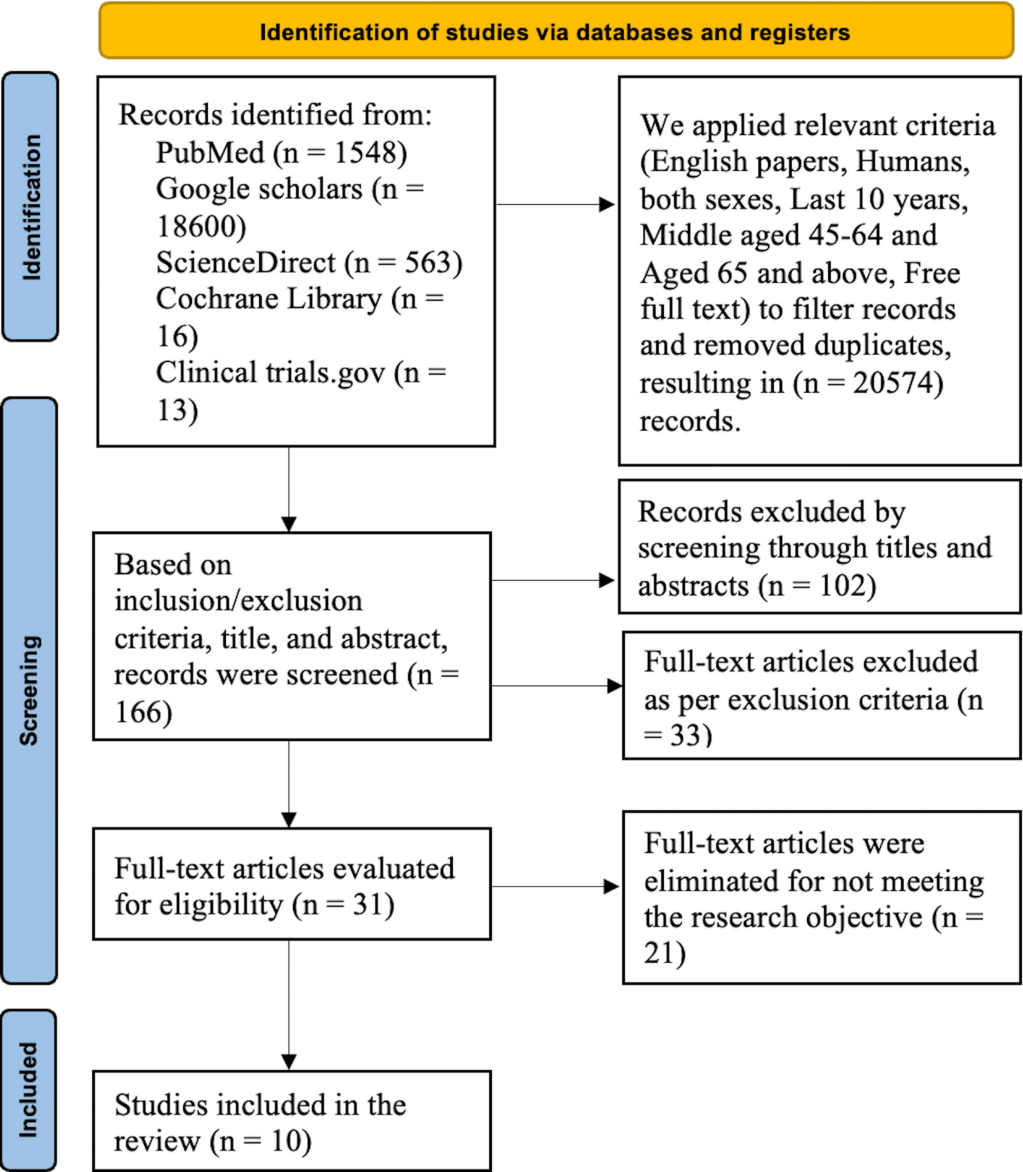
PRISMA flow diagram PRISMA: Preferred Reporting Items for Systematic Reviews and Meta-Analyses

We conducted a comprehensive research analysis on high-fiber PBDs and their impact on parameters that contribute to CVD risk factors. Out of a total of 20,740 papers, we found 10 articles that contained the complete text. Using filters such as English language, human studies, and publications from the past decade, we excluded 20,574 papers. After evaluating titles and abstracts, 102 articles were eliminated from consideration. Additionally, 33 papers were excluded for failing to meet the exclusion criteria. Subsequently, 21 publications were excluded because they did not correspond with the research objectives, resulting in a final selection of 10 papers. The investigations comprised three systematic reviews, two observational studies, four review articles, and one RCT involving many patients with different CVD risk factors. The study selection process is depicted in the PRISMA flow chart (Figure 1).

The data extraction process was carried out after a thorough examination for any possible bias. The intervention examined, the participant count, the study design, the outcome, and the conclusion were documented and scrutinized for subsequent deliberation, as depicted in Table 6.

**Table 6:** Summary of included articles

Author and year of publication	Intervention examined	Participant count	Study design	Outcome		Conclusion
Gan et al. (2021) [17]	Plant-based dietary patterns	A total of 698,707 participants, out of which 137,968 experienced cardiovascular disease (CVD), 41,162 had coronary heart disease (CHD), and 13,370 had stroke episodes.	Systematic Review and Meta-Analysis of Prospective Cohort Studies	The risk of CVD (relative risk (RR): 0.84; 95% confidence interval (CI): 0.79-0.89) and CHD (RR 0.88; 95% CI: 0.81-0.94) was lower in those who followed the plant-based diets the most, but not for stroke (RR: 0.87; 95% CI: 0.73-1.03). Adherence to the plant-based diet index (PDI) was assessed, and a correlation was discovered with a reduced risk of CVD.		Plant-based diets are characterized by increased consumption of plant foods and decreased consumption of animal foods. These diets are effective for preventing CVD.
Austin et al. (2021) [18]	Plant-based diets (PBDs) vs. regular meat-eating diets (RMDs)	269	Randomized Controlled Trials (RCTs): a systematic review and meta-analysis	The intake of PBDs led to a significant reduction in body weight (-2.35 kg, 95% CI: -3.51, -1.19, p < 0.001), body mass index (BMI) (-0.90 kg/m^2^, 95% CI: -1.42, -0.38, p = 0.001), and waist circumference (WC) (-2.41 cm, 95% CI: -3.72, -1.09, p < 0.001).		PBDs have the potential to improve the management of central adiposity because they are effective in lowering body weight, BMI, and waist circumference in people with type 2 diabetes mellitus (T2DM).
Yao et al. (2023) [19]	Consumption of vegetables, fruits, cereals, as well as soluble and insoluble fiber in one's diet	2,567,890	Systematic review and meta-analysis	Summary relative risk for mortality from all causes is 0.90 (95% CI: 0.86-0.93); for mortality from CVD, it is 0.87 (95% CI: 0.84-0.91).		Increased consumption of dietary fiber is linked to a decreased likelihood of death from any cause, cardiovascular diseases, and cancer.
Wright et al. (2017) [20]	Whole food plant-based (WFPB) diet	65	Randomized controlled trial (RCT)	There was a notable decrease in BMI by 4.4 kg/m² and cholesterol by 0.71 mmol/L after six months.		WFPB diet leads to significant improvements in BMI, cholesterol, and other risk factors.
Soliman (2019) [21]	The impact of dietary fiber intake on atherosclerosis and cardiovascular disease risk	Not specified	Review article	Consuming dietary fiber is linked to a reduced likelihood of developing cardiovascular disease. Soluble fibers lower blood cholesterol, and insoluble fibers promote digestive regularity by decreasing intestinal transit time and increasing fecal bulk.	Increasing dietary fiber intake, both soluble and insoluble, is crucial for reducing cardiovascular disease risk. When added to food during processing, functional fibers also have beneficial health effects.	
Nepali et al. (2022) [22]	Dietary fiber	Not specified	Review article	Increased consumption of dietary fiber is linked to decreased blood pressure and enhanced cardiovascular well-being.	Dietary fibers play a significant role in managing hypertension and reducing cardiovascular risk factors.	
Thomas et al. (2022) [23]	The impact of healthy PBDs on metabolic syndrome (MetS) parameters, including dyslipidemia, insulin resistance, and inflammation	Not specified	Narrative review	PBDs are associated with lower risk factors for MetS, such as lower plasma triglycerides, total cholesterol, low-density lipoprotein (LDL) cholesterol, fasting blood glucose, blood pressure, and inflammation.	Healthy plant-based diets improve metabolic syndrome parameters, including dyslipidemias, insulin resistance, and inflammation. Different types of PBDs offer varied benefits and the quality of diet matters.	
Salehin et al. (2023) [24]	Plant-based diet vs. conventional diet	Not specified	Review article	Multiple studies indicate that adopting a diet primarily composed of plant-based foods decreases the risk of death from any cause, mortality related to ischemic heart disease (IHD), improves blood pressure and glycemic and lipid regulation, and reduces the need for prescription drugs.	Plant-based diets are linked with significant cardiovascular benefits, including lower mortality and better management of cardiovascular risk factors. This review supports dietary interventions as an effective strategy for cardiovascular disease prevention and management.	
Zhang et al. (2022) [25]	Association between dietary fiber intake and long-term CVD risk	14,947	Cross-sectional Study	Participants at intermediate and high risk who had a higher density of dietary fiber in their diet were found to have a lower risk of atherosclerotic cardiovascular disease (ASCVD). Additionally, high-risk participants who consumed a higher amount of total dietary fiber also had a reduced risk of ASCVD.	Participants at intermediate and high risk who adhered to a diet rich in dietary fiber had a reduced likelihood of developing ASCVD. Additionally, high-risk participants who consumed a larger amount of total dietary fiber had a reduced risk of ASCVD.	
Shivakoti et al. (2022) [26]	Total fiber content and fiber sources (fruit, vegetable, cereal)	4,125	Cohort study	Lower inflammation was linked to higher intakes of both total and cereal fiber. Inflammation contributed roughly 16% of the negative association between cereal fiber and CVD.	Increased consumption of cereal fiber is linked to reduced inflammation and a decreased risk of CVD, suggesting a modest role for inflammation as a mediator.	

Discussion

Impact of High-Fiber PBDs on CVD Risk Factors

Researchers have conducted multiple studies on the cardiovascular health benefits of high-fiber PBDs. The discussion combines evidence from numerous pivotal studies that show the impact of these diets on CVD risk factors, such as cholesterol levels, blood pressure, and indicators of atherosclerosis.

**Figure 2:**
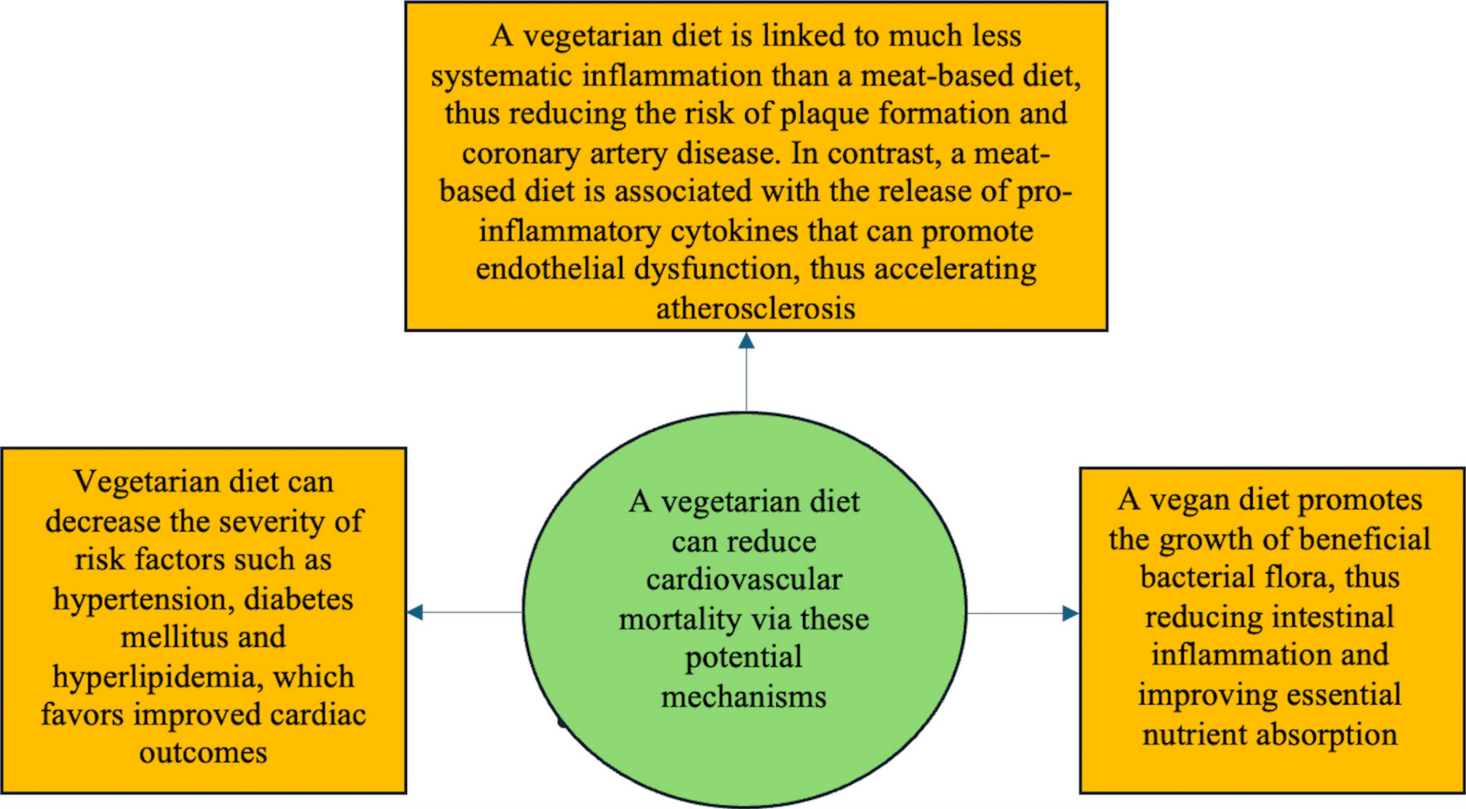
Potential mechanisms that elucidate the positive cardiovascular results of a vegetarian or vegan diet Adapted from Salehin et al. (2023) [24]

Salehin et al. conducted a compelling study on the impact of PBD patterns on CVD risk. Their findings indicated that individuals who adhered to PBDs experienced significantly better health outcomes than those following a typical diet. A critical factor in this difference was the higher proportion of heme iron from meat, known for its inflammatory properties, compared to the non-heme iron found in vegetables. The study suggests that prolonged exposure to pro-inflammatory chemicals in the bloodstream increases susceptibility to coronary atherosclerotic disease and adverse cardiac events. Research has shown that diets high in processed meats, dairy, and other animal products can elevate pro-inflammatory markers in the body, contributing to endothelial dysfunction - the impairment of the cell layer lining blood vessels. This chronic inflammation, marked by elevated circulating C-reactive protein (CRP) and other inflammatory substances, facilitates plaque formation, leading to more severe cardiovascular issues [24]. Moreover, endothelial dysfunction exacerbates atherosclerosis by promoting platelet aggregation, increasing endothelial permeability, stimulating cytokine production, and enhancing white blood cell adhesion [27]. This study underscores the cardioprotective benefits of PBDs and highlights the need for further research to understand how dietary patterns influence inflammation and cardiovascular health (Figure 2).

Nepali et al. evaluated dietary fiber's impact on managing hypertension, a significant risk factor for CVD. According to their research, nutritional fibers are plant components that cannot be broken down or absorbed in the human small intestine. Instead, they are fully or partially fermented in the large intestine. The study highlighted that enterocytes use butyrate, one of the SCFA byproducts of fermentation, as an energy source to maintain gastrointestinal cell integrity. Some of these byproducts indirectly stimulate the growth of other microbes, thus modulating the human gastrointestinal microbiota. It also suggested that a decrease in levels of SCFA-producing bacteria and a drop in butyrate levels in the gastrointestinal microbiota lead to gut dysbiosis, which is associated with hypertension [22]. These findings emphasized that dietary modifications modulating gut microbiota are essential in managing hypertension. 

Shivakoti et al. explored the intriguing link between dietary fiber consumption and general markers of inflammation, such as CRP, interleukin-6 (IL-6), and tumor necrosis factor-alpha (TNF-α), in both healthy individuals and those with metabolic diseases. Their study revealed a compelling negative relationship between overall fiber intake and inflammation markers, highlighting the anti-inflammatory potential of fiber. Interestingly, the source of fiber played a crucial role in this association. Cereal fiber, rather than vegetable or fruit fiber, significantly impacted inflammation levels. Specifically, they observed a negative correlation between total fiber and cereal fiber intake with IL-1 receptor antagonist (IL-1RA), an anti-inflammatory cytokine that blocks pro-inflammatory interleukins by binding to their receptors.

This finding underscores the broader anti-inflammatory properties of dietary fiber, which can enhance lipid and glucose metabolism, improve gut function, and influence diet and satiety by reducing fat and total energy intake. However, the precise mechanisms behind the unique anti-inflammatory effects of cereal fiber, as opposed to other fiber types, remain a mystery and warrant further investigation [26]. Their study opens the door to a deeper understanding of how different fiber sources can differentially impact inflammation and metabolic health, highlighting the need for more targeted research in this area.

Comparative of the Performance of PBDs With Alternative Dietary Interventions

PBDs are attracting significant popularity for their potential benefits in controlling multiple illnesses, including obesity, ischemic heart disease, and diabetes. These articles analyze the success rate of PBDs compared to other dietary interventions, specifically concerning how they impact the regulation of weight and cardiovascular health in people with T2DM and other metabolic disorders.

Austin et al. performed a thorough examination and meta-analysis of RCTs to examine the impact of PBDs on the overall body weight of individuals with T2DM. The study revealed that individuals who strictly followed PBDs had more substantial decreases in weight, BMI, and waist circumference (WC) compared to those who stuck to regular meat-eating diets (RMDs). The study proposed that a duration of at least 16 weeks, with no restriction on energy consumption, successfully decreased body weight and BMI. Vegan diets successfully lower BMI and body weight. The decrease in body weight was primarily associated with the abundant fiber content, low-calorie density, and increased consumption of whole foods, such as fruits, vegetables, and legumes. Moreover, a robust correlation exists between diets mainly composed of vegetables and fruits and improved regulation of glucose levels, as seen by decreased hemoglobin A1c levels [18]. This study may offer data to endorse the adoption of vegan dietary patterns to aid in the more effective control of body weight in people with T2DM.

Wright et al. conducted a community-based RCT involving individuals with diabetes, ischemic heart disease, or obesity who adopted a WFPB diet without requiring regular exercise or calorie restriction. The trial's findings were striking, revealing that participants who embraced the PBD experienced significantly greater reductions in BMI and WC than those following conventional dietary guidelines. This dietary shift promoted weight loss and improved lipid profiles, as evidenced by decreased total cholesterol and low-density lipoprotein (LDL) cholesterol levels, which are crucial for reducing the risk of cardiovascular events among individuals with ischemic heart disease and diabetes. Participants reported high satisfaction and adherence to the PBD, highlighting its potential for long-term success in weight management and chronic disease prevention. In contrast, other dietary interventions often struggle with sustainability and adherence due to their restrictive nature [20]. This study underscores the transformative power of a WFPB diet, offering a sustainable and practical approach to improving health outcomes in vulnerable populations.

Overall, the comparative effectiveness of PBDs versus other dietary interventions highlights their significant benefits due to their high fiber content and nutrient density. PBDs not only facilitate weight loss and enhance glycemic control in persons diagnosed with T2DM but also offer cardiovascular benefits by reducing cholesterol levels and inflammation. These diets provide a comprehensive approach to managing obesity, ischemic heart disease, and diabetes, making them a valuable dietary strategy for enhancing general well-being and minimizing the likelihood of chronic illnesses. Future research should explore PBDs' long-term effects and adherence strategies to establish their role in clinical and community settings.

How High-Fiber PBDs Improve Cardiovascular Health

Many studies have shown that consuming diets high in plant-based foods and fiber can significantly improve cardiovascular health through various processes. This discussion summarizes recent studies to explain how these diets improve cardiovascular outcomes.

Improvement of dyslipidemias, insulin resistance, and inflammation: Gan et al. investigated the impact of plant-based foods, which are low in energy density but rich in fiber, vitamins, and phytonutrients, on various health outcomes. They discovered that such diets significantly improve weight management, glycemic control, lipid levels, blood pressure, and inflammation - all crucial in preventing atherosclerosis. The study highlighted that gut bacteria convert choline and L-carnitine from animal-based diets into TMAO, a compound that promotes atherosclerosis and increases CVD risk. PBDs are abundant in soluble and insoluble fibers found in vegetables, fruits, legumes, and whole grains. Like those in oats and beans, soluble fibers lower blood cholesterol by binding to bile acids and facilitating their elimination, thereby reducing LDL cholesterol - a key contributor to atherosclerosis. High-fiber diets also enhance insulin sensitivity and lower cholesterol levels by slowing carbohydrate absorption, preventing sudden blood sugar spikes, and improving overall glucose metabolism. Additionally, these diets are rich in antioxidants and phytochemicals that combat inflammation, a significant factor in CVD development [17]. This research underscores the holistic benefits of PBDs for cardiovascular health.

Reduction of atherosclerosis: Salehin et al.'s study showed how diets high in dairy, processed meats, and other animal products have been linked to pro-inflammatory indicators that promote endothelial dysfunction. This dysfunction, in turn, encourages the development of atherosclerosis by causing platelet aggregation, increased permeability of the endothelium, production of cytokines, and adhesion of leukocytes. Ultimately, these factors lead to a deterioration in cardiac health. Nevertheless, a diet mainly consisting of plants is linked to significantly lower systemic inflammation levels than a diet centered around meat consumption. Consequently, this reduces the likelihood of plaque buildup and the development of coronary artery disease [24].

Impact on cardiovascular and cancer mortality: Soliman emphasizes that dietary fiber protects against chronic illnesses such as CVD, diabetes, metabolic syndrome, inflammatory bowel syndrome, diverticular disease, obesity, and colon cancer, as shown in the analysis adjusted for age. It mentions that insoluble fiber binds to and absorbs carcinogens, mutagens, and toxins, and, therefore, prevents their harmful effects on the body by preventing the absorption of these toxins and targeting them for elimination. This study investigates additional fiber qualities, such as the effect on the time it takes for food to pass through the colon, the lengthening of the feeling of fullness and satisfaction after a meal, and the stimulation of the cholecystokinin hormone that promotes satiety. Additionally, it suggests that incorporating dietary fiber into one's diet can be a beneficial modification to accompany the use of statin monotherapy to reduce overall and LDL cholesterol levels, lower the required dosage of statin medication, minimize adverse effects, and enhance the body's ability to tolerate the medicine [21].

In their study, Yao et al. found a correlation between the consumption of dietary fiber and mortality rates from different causes, such as CVD and cancer. They also examined the relationship between the intake of various sources and types of fiber and mortality. The study showed that dietary fiber dramatically lowered postprandial glucose and insulin responses. This inhibits the hepatic enzyme 3-hydroxy-3-methylglutaryl-coenzyme A (HMG-CoA) reductase, preventing the liver from synthesizing and releasing excess cholesterol into the bloodstream, which potentially lowers the risk of CVD. Moreover, a reverse correlation was discovered between all fiber types and CVD mortality. However, a negative correlation between fiber and cancer mortality was observed only for dietary and cereal fiber.

Additionally, the study highlighted that overall fiber consumption correlated with lower overall mortality, excluding fiber from fruits and vegetables. A meta-analysis of five trials revealed that consuming 25 to 29 grams of fiber per day was associated with the most significant reduction in all-cause mortality, with benefits potentially increasing beyond 30 grams daily. Interestingly, the study showed a nonlinear relationship between fiber intake and all-cause mortality, indicating varying health benefits at different intake levels. However, this nonlinear pattern was not seen for cancer mortality, suggesting distinct mechanisms by which fiber influences health outcomes [19]. Yao et al. emphasize the need for further research to explore the specific impacts of soluble and insoluble fiber and the effects of various fiber sources on cancer mortality.

Ultimately, plant fiber diets benefit cardiovascular health by lowering dyslipidemias, increasing insulin sensitivity, reducing inflammation, preventing atherosclerosis, and improving metabolic health. These dietary plans offer a successful strategy to prevent cardiovascular problems while improving long-term health outcomes. More research is necessary to investigate the specific types and origins of dietary fibers that have the most beneficial impact on cardiovascular health, as well as the long-term strategies to maintain these favorable nutritional patterns.

Limitations

Despite the compelling evidence supporting the health benefits of high-fiber PBDs, it is important to acknowledge several limitations. Many studies relied on self-reported dietary intake, which can introduce bias and inaccuracies. Additionally, there is a need for more research on the long-term adherence and sustainability of PBDs across diverse populations. While we observed a statistically insignificant yet potentially protective trend for stroke with high-fiber diets, the variation in research settings contributes to these inconsistencies. Further investigations are necessary to identify specific types and sources of dietary fibers that have the most beneficial impact on cardiovascular health and to develop strategies for maintaining these dietary patterns over the long term.

## Conclusions

In conclusion, our systematic review underscores the substantial benefits of PBDs in mitigating CVD risk factors. High-fiber PBDs are linked to increased insulin sensitivity, lower total and LDL cholesterol levels, and reduced inflammation, which diminish atherosclerosis progression and CVD incidence. Our findings reveal that dietary fiber enhances cardiovascular health and reduces mortality from CVD and cancer, making it a valuable non-pharmacological intervention. These insights highlight the critical public health implications of shifting dietary patterns, which include reducing animal products and unhealthy plant foods while increasing high-fiber plant foods to improve cardiovascular health. Although further research is needed to identify the most effective types of dietary fiber, promoting dietary fiber intake across different age groups can significantly improve cardiovascular health outcomes. Emphasizing dietary fiber in public health strategies could pave the way for more effective prevention and management of CVDs.
